# Implementation of highly challenging balance training for Parkinson’s disease in clinical practice: a process evaluation

**DOI:** 10.1186/s12877-021-02031-1

**Published:** 2021-02-01

**Authors:** Breiffni Leavy, Conran Joseph, Lydia Kwak, Erika Franzén

**Affiliations:** 1 Department of Neurobiology, Care Sciences and Society, Division of Physiotherapy, Karolinska Institutet, Huddinge, Sweden; 2 The Stockholm Sjukhem Foundation, Stockholm, Sweden; 3 Department of Health and Rehabilitation Sciences, Division of Physiotherapy, Stellenbosch University, Cape Town, South Africa; 4 Unit of Intervention and Implementation Research for worker health, Institute of Environmental Medicine, Karolinska Institutet, Stockholm, Sweden; 5 Function Area Occupational therapy & Physiotherapy, Allied Health Professionals Function, Karolinska University Hospital, Stockholm, Sweden

**Keywords:** Process evaluation, Balance training, Parkinson’s disease

## Abstract

**Background:**

Process evaluations provide contextual insight into the way in which interventions are delivered. This information is essential when designing strategies to implement programs into wider clinical practice. We performed a process evaluation of the HiBalance effectiveness trial investigating the effects of a 10-week of highly challenging and progressive balance training for mild-moderate Parkinson’s disease (PD). Study aims were to investigate i) the quality and quantity of intervention delivery and ii) barriers and facilitators for implementation.

**Methods:**

Process outcomes included; Fidelity; Dose (delivered and received) Recruitment and Reach. Investigation of barriers and facilitators was guided by the Consolidated Framework for Implementation Research. Program delivery was assessed across four neurological rehabilitation sites during a two-year period. Data collection was mixed-methods in nature and quantitative and qualitative data were merged during the analysis phase.

**Results:**

Thirteen program trainers delivered the intervention to 12 separate groups during 119 training sessions. Trainer fidelity to program core components was very high in 104 (87%) of the sessions. Participant responsiveness to the core components was generally high, although adherence to the home exercise program was low (50%). No significant context-specific differences were observed across sites in terms of fidelity, dose delivered/ received or participant characteristics, despite varying recruitment methods. Facilitators to program delivery were; PD-specificity, high training frequency and professional autonomy. Perceived barriers included; cognitive impairment, absent reactional balance among participants, as well a heterogeneous group in relation to balance capacity.

**Conclusion:**

These findings provide corroborating evidence for outcome evaluation results and valuable information for the further adaptation and implementation of this program. Important lessons can also be learned for researchers and clinicians planning to implement challenging exercise training programs for people with mild-moderate PD.

**Trial registration:**

ClinicalTrials.gov, NCT02727478, registered 30 march, 2016 − Retrospectively registered.

**Supplementary Information:**

The online version contains supplementary material available at 10.1186/s12877-021-02031-1.

## Background

Once the efficacy and effectiveness of a healthcare intervention has been established the next step is to implement findings in order to reach typical patients [1]. A process evaluation is considered a key component in making this transition from research knowledge into standard clinical practice [2]. Balance training interventions for people with Parkinson’s disease (PwPD) have proven efficacy in terms of symptom management [3–5]. Although the number of intervention trials of exercise interventions in PD has grown exponentially [6], process evaluations in the field of neurological rehabilitation are less frequent [7]. There is therefore, a lack of scientific evidence to guide researchers and clinicians towards dissemination of effective programs. Important lessons for implementation can be learned by investigating the quality and quantity of the intervention delivered, and such information can strengthen confidence in the conclusions drawn from the outcome evaluation [2, 8]. Additionally, by analyzing the work and experiences of clinicians who delivered an intervention, process evaluations assess the generalizability of the program within the clinical context [2].

We have previously established the efficacy and effectiveness of the HiBalance program [9, 10] − a 10-week highly challenging and progressive balance group training for PwPD. Findings from the outcome evaluation of this intervention have been published but to summarize, significant improvements were seen in balance, gait speed and dual-task functional mobility among the training group compared to controls [10]. Improvement occurred in two of four balance sub-domains of the MiniBESTest, the primary outcome measure.

This study is the process evaluation of the clinical effectiveness trial, which was performed concurrently as part of a type 1 hybrid effectiveness-implementation design [11], as previously outlined in the full trial protocol [12]. The aims of this process evaluation were to; i) examine the quality and quantity of the HiBalance program as it was delivered across four neurological rehabilitation sites by assessing Fidelity, Dose (delivered and received), Recruitment and Reach and ii) investigate barriers and facilitators to implementation in outpatient care.

## Methods

### Study design

This study was guided by Medical Research Council (MRC) guidelines, which provide a framework for conducting process evaluations [2]. Measures used to support the implementation process included; shared decision-making between the research group and clinicians; Training of physical therapist clinicians at each respective site; Development and revision of standardized program materials (Additional file 1) and ongoing support throughout the trial when requested by clinicians. The study has a triangulation mixed-method design [13], whereby qualitative and quantitative data were collected concurrently, then interrelated during the analysis to inform one interpretation. The epistemological grounds of mixed-methods research is pragmatism, whereby a focus on the research question determines the nature of the data to be collected and merged [14]. The use of theory to guide and inform process evaluations is recommended [7]. The Consolidated Framework for Intervention Research (CFIR) [15] guided the investigation of barriers and facilitators to HiBalance implementation. CFIR provides a ‘menu’ of operationally described constructs − determinants that support or hinder implementation success. This study adheres to the CONSORT guidelines for trial reporting [16].

### HiBalance program core components and causal mechanisms

The primary hypothesis of the HiBalance program was that highly challenging, specific and progressive balance training would improve balance and gait in PD. Program core components were grounded on established principles of exercise; i) *Overload of balance challenge*: that the balance exercises should be performed at an intensity level which challenged participants at the limits of their capacity, in order to facilitate physiological adaptation [17]. Trainers encouraged this principle by continuously adapting exercises to each participant’s capacity in order to elicit reactive postural responses during the sessions; ii) *Specificity:* physical adaptation is specific to the type of training undertaken. Specificity was incorporated by separately targeting four balance sub-components impaired in Parkinson’s disease, two per session on alternate weeks. iii) *Progression:* placement of a gradual increase in stress on the body to force adaptation to changing stimuli. Systematic progression of the complexity of group exercises was outlined in a 10-week framework (previously published) [10]. The balance sub-components challenged included, *Sensory integration*, targeted due to difficulties for PwPD to integrate peripheral sensory stimuli necessary when predicting a motor response. Withdrawal of visual feedback while walking on varied surfaces was an example of a progressed exercise in this balance sub-component. *Anticipatory postural adjustments* were targeted in order to train the postural muscle activation responses which occur prior to a voluntary movement in order to counteract balance destabilization. Sit to stand exercises and movements in standing while throwing and catching a ball, were examples of exercises in this sub-component. *Stability limits* were challenged during for example, reaching exercises where participants were required to move their center of mass, without adjusting their base of support in standing. *Motor agility* was targeted through exercises involving reciprocal and whole-body coordination during different walking conditions. Participants also trained quick shifts in speed, amplitude and direction of the movements during gait. Additionally, we hypothesized that gradual exposure to cognitive and motor dual-task conditions would improve dual-task performance, and that addition of a home exercise program (HEP) − which focused on lower limb and core-strengthening exercises − would improve participant’s capacity to partake in the hour long clinical training sessions and increase their physical activity level. Participants were encouraged to perform the HEP a minimum of once per week, which incorporated six functional strength training exercises and 30 min (achievable through 10 min bouts) of pulse-increasing exercise such as walking or cycling on an exercise bike. Nonetheless, the HEP was considered an adaptive intervention component, as modification of the HEP was not anticipated to significantly alter intervention effects.

### Data collection

#### Process evaluation outcomes (quantitative data)

*Fidelity* involves the extent to which core components, or essential elements, of the program were delivered as intended by program developers [18]. Analysis of fidelity allows judgement of whether the outcomes achieved in a trial were due to that which was delivered. Assessed components of fidelity included i) *quality of delivery of program core components* and ii) *Participant responsiveness –* the extent to which the target audience engages with or is satisfied with the intervention [18]. Trainers documented their adherence to program core components using standardized training report cards describing the type of exercises, as well as the balance sub-components and/or dual-task exercise performed during each session. *Participant responsiveness* to the core components –overload of balance challenge, progression and specificity − was investigated using a self-administered questionnaire. This structured questionnaire was developed in order to capture participant experiences of specific aspects of the program and has been published in the study protocol [12]. *Dose delivered* is the frequency of intervention provided by program trainers. *Dose received* is the frequency of intervention received by PwPD, this included the twice-weekly clinical training sessions and the once-weekly HEP. This data was collected using standardized protocol forms and patient questionnaire respectively. *Recruitment* refers to the procedure used to engage rehabilitation clinics and PwPD. In the present study, each clinic was encouraged to recruit independently and to register logs concerning the process. Descriptive characteristics of participating clinics, as well as figures for patient turnover at each primary/out-patient care department, were gathered through email contact with clinic administrative controllers. In implementation research *Reach* relates to the extent to which the target audience comes into contact with the intervention. In the present study however, it was not our intention to reach all PwPD in the Stockholm region. Reach was therefore operationalized as the variation in participant characteristics across training sites.

#### Barriers and facilitators (qualitative data)

Focus group interviews were used to explore trainer perceptions of the program and potential barriers and facilitators to program implementation, as well as capture their suggestions for eventual adaptation. The interview guide, previously published in the study protocol [12], was developed in accordance with CFIR guidelines, whereby interview questions were linked to CFIR domains. We followed MRC recommendations by; i) collecting data at multiple time points to capture eventual changes in perceptions over time and ii) including a smaller selection of program implementers. All trainers were invited via mail to take part in the interviews, participation was voluntary, but all clinics were advised to be represented. Focus group interviews were moderated by the author BL and one interview assisted by a doctoral student. The interview location rotated between participating clinics. Three interviews were performed at 6 − month intervals over a one-and-a-half-year period. This amounted to 270 min of digital recording, which was transcribed verbatim. Open-ended interview questions were based on CFIR constructs considered relevant and the interview guide and corresponding constructs are previously published [12]. Qualitative data were collected iteratively so that if a theme emerged in the first focus group interview one, it was followed up in successive interviews.

### Data Analysis

#### Quantitative data

*Quality of delivery of the core components* was analyzed through a comparative analysis of each training report card with the HiBalance 10-week structural framework. Firstly, a research assistant with program experience analyzed each outlined exercise regarding the balance sub-components targeted and categorized whether the exercise targeted the correct balance subdomain as i) yes, ii) no and iii) partially. These categorizations were then independently checked at a second stage by the author BL, documented and summarized using Microsoft Excel. *Participant responsiveness* data from the questionnaire were coded in Excel and analyzed using descriptive statistics. *Dose delivered* and *received data* were extracted from protocol and planning forms and entered into Excel spreadsheets before being summarized statistically. In relation to reach, patient characteristics at baseline were summarized and between-clinic variation in patient groups was analyzed using analysis of variance (ANOVA) test for normally distributed data and *Pearson’s* Chi-squared test was used for non-parametric analysis of variance. All statistical analysis was performed using *Stata Statistical Software: Release 15*, College Station, TX. Statacorp LLC.

#### Qualitative data

Interview transcripts were analyzed using the qualitative analysis approach − Directed content analysis [19]. The distinguishing feature of this method is that initial coding is guiding by existing theory regarding the phenomenon – a relevant approach due to our application of CFIR constructs when exploring determinants of implementation. Steps taken in the analysis are summarized by Mayring [20], and firstly involve a structured pre-definition of initial categories based on theory. After thorough reading of the transcripts, sections of the text were systematically assigned to pre-defined CFIR codes in a process of ‘deductive category application’ [20]. The predefined codes were based on the constructs *Intervention Characteristics*, *Outer setting* and *Characteristics of Individuals*. Meaning units were grouped together using an organizational matrix in Microsoft Excel, where similar codes were grouped to form categories. These categories were then further interpreted, with respect to whether they represented barriers or facilitators to program implementation, in peer-debriefing sessions involving authors BL and CJ.

## Results

Between March 2016 and Feb 2018, a total of thirteen program trainers (Table 1) across four clinics, delivered the intervention to 12 separate groups including a total of 61 PwPD. In total, 119 supervised training sessions were performed (dose delivered), as one training session was cancelled at one clinic during the study period.

**Table 1:** Characteristics of trainers who participated in the study

Age group	Sex	Clinic	Professional Experience (Years)	Neurology Experience (Years)	Previous experience of program (Y/N)	Number of groups trained	Participated in focus group interviews
30–34	F	1	4	2	Y	1	Y
45–49	M	1	m	m	Y	1	N
25–29	F	1	2	2	N	1	Y
30–34	F	1	6	6	N	1	N
35–39	F	2	11	10	Y	4	Y
45–49	F	2	22	18	Y	4	Y
25–29	F	2	3	2	N	1	Y
40–44	M	3	13	10	N	1	N
65–69	F	3	37	26	N	1	Y
35–39	F	3	4	4	N	1	Y
50–54	F	3	7	.5	N	1	N
50–54	F	4	18	18	N	3	Y
50–54	F	4	16	16	N	3	Y

*Abbreviations*: *M* male, *F* female, *Y* yes, *N* no, *m* missing

### Fidelity

#### Quality of delivery of the program core components

Training planning and report protocols from 110 group-training sessions were analyzed, data from nine sessions (7% of total dose delivered) at one clinic were missing. Overall trainer fidelity to the core components of the group-training sessions was very high. In 104 out of 119 (87%), of the group training sessions, trainers designed exercises, which adhered to the 10-week HiBalance framework that outlines the specific balance sub-domains and dual-task components to be targeted, during which weeks. In the five sessions (4%) where fidelity was poor-fair, balance subdomains were trained, but exercises were not exactly in line with the 10-week HiBalance scheme. These five sessions all occurred at one clinic. We also analyzed the nature of the exercise focus in Block C (weeks 7–10), where trainers were free to choose among the four balance components to target. In three of the four clinics, there was a tendency for trainers to continue to follow the training protocol scheme from Block B by focusing on two specific balance components per week ─ *Stability Limits* and *Motor Agility* during week 7, and *Anticipatory Postural Adjustments* and *Sensory Integration* during week 8 ─ nevertheless, motor and cognitive dual task exercises were integrated freely as advised. In the final two weeks of the program, all HiBalance subdomains were targeted within the same training sessions at all clinics.

#### Participant responsiveness to the core components

A majority (71%) of participants responded positively to the *Overload* of balance challenge component of the program – by responding that their balance was challenged to ‘partially’ or to ‘a high’ degree (See Table 2). Similarly, regarding perceptions of the *Progressive* nature of program difficulty, 88% of respondents reported that balance challenge had been progressed either partially/ to a high or very high degree. Program *Specificity* was explored in relation to perceived difficulty of exercises targeting the four specific balance sub-domains. Participants reported, for example, that exercises targeting *Sensory integration* as the most challenging and exercises targeting *Anticipatory Postural adjustment* as the least challenging of the four domains.

**Table 2:** Participant responsiveness to core components of the HiBalance intervention

Participant perceptions	To a very small degree n (%)	To a Small degree n (%)	Partly n (%)	To a high degree n (%)	To a very high degree n (%)	Do not know n (%)
**Overload of balance challenge** My balance was challenged during group training	*3 (6)*	*12 (23)*	*35 (67)*	*2 (4)*	*–*	–
**Training progression** The difficulty level increased during the training period	*1 (2)*	*5 (10)*	*25 (48)*	*19 (37)*	*2 (4)*	–
**Training specificity** **i) Stability Limits** Exercises involving trunk rotations and controlled leaning exercises challenged my balance	*3 (6)*	*13 (27)*	*17 (35)*	*14 (29)*	*1 (2)*	*1 (2)*
**ii) Anticipatory Postural Adjustments** Exercises involving kicking and throwing a ball challenged my balance	*5 (10)*	*12 (24)*	*17 (35)*	*12 (24)*	*2 (4)*	*1 (2)*
**iii) Sensory Integration** Exercises involving standing on soft and unstable surfaces challenged my balance	*1 (2)*	*3 (6)*	*16 (30)*	*23 (43)*	*10 (19)*	*–*
**iv) Motor Agility** Exercises involving walking over and around obstacles challenged my balance	*2 (4)*	*9 (17)*	*16 (30)*	*21 (40)*	*5 (9)*	*–*
**Motor dual-task exercises** To what extent did the added motoric task (e.g balancing items on a tray while walking) challenge your balance?	*–*	*2 (4)*	*19 (36)*	*26 (49)*	*6 (11)*	*–*
**Cognitive dual-task exercises** To what extent did the added cognitive tasks (e.g.counting numbers) challenge your balance?	*2 (4)*	*13 (25)*	*22 (42)*	*12 (23)*	*4 (8)*	*–*

### Dose received

Overall, 85% (SD: 11) of training sessions were attended by participants. The range for attendance as per the predetermined 20 sessions was 50–100%. A further sub-analysis showed that 12 participants attended between 50 to 75% (10 to 15 sessions), whereas 41 participants attended between 80 and 100% (16 to all 20 sessions). The ANOVA test indicates no differences with respect to attendance rates across the four training clinics (Table 3).

**Table 3:** Dose received among those who completed the training program (*n* = 53)

Clinic	Participants trained	Mean attendance, % (SD)
1	*n* = 12	82% (11.7)
2	*n* = 20	84% (12.0)
3	*n* = 7	84% (8.9)
4	*n* = 14	90% (8.9)

In terms of the HEP, approx. 50% of participants reported completing it, and a majority (*n* = 47 (77%) indicated that the individual exercises in the HEP were adapted to their ability at training outset. A further 77% of participants indicated that they were motivated to continue with the HEP once the training ended.

### Recruitment

#### Recruitment of rehabilitation clinics where training occured

The research group selected eight clinics of varying nature and geographical location providing rehabilitation in the Stockholm region (Additional file 2). Clinics were approached firstly by mail, and subsequently by telephone if interest was expressed in the study. Six out of the eight clinics contacted agreed to join the study. Four clinics joined as ‘training clinics’ and provided the HiBalance intervention. Clinics were included consecutively, with training commenced at clinics 1 and 2 during Spring 2016 and at clinics 3 and 4 during Autumn 2016.

#### Recruitment of study participants at the clinics

Each clinic was encouraged to recruit patients according to local routines. The recruitment process varied dependent on the clinics previous experience providing specialized neurological and/or PD outpatient group training (See Additional file 2). Clinic four, a specialized neurological clinic with in- and out-patient rehabilitation, recruited independently to all groups through a process of internal referral. Clinic three on the other hand − a geriatric rehabilitation hospital with no previous experience of specialized out-patient PD group training − relied entirely on advertisement in local newspapers for recruitment. The research group assisted in this process. Clinics one and two, both with previous experience of PD-specific group training, initially intended to recruit from internal waiting lists, but were required to use advertisement when numbers were insufficient to fills groups. Inability of clinics one and two to recruit 12 participants respectively prior to study start resulted in insufficient numbers to enable randomization, resulting in a non-randomized study design. Higher staff turnover of trainers at clinic one & three resulted in fewer groups being trained at these sites, as time was required to recruit and educate new trainers.

### Reach

Parametric analysis of variance of the baseline characteristics of PwPD at the various clinics showed no significant differences between participants recruited in relation to; age (*P* = 0.165), years with the disease (*P* = 0.695), balance control (*P = 0.648),* gait speed (*P* = 0.688) or physical activity level (*P* = 0.132). The pattern was similar for the variance of non-parametric data; Functional mobility (chi-squared: 4.45, *P* = 0.216) and Executive function (chi-squared: 5.77, *P* = 0.122). Different recruitment processes did not therefore result in different samples of PwPD in relation to descriptive or disease-related characteristics. We consider that our target group of those with mild-moderate PD came into contact with the intervention at all training sites.

#### Response to the training program at different sites

No statistical difference was found in the proportion of those who improved their balance (beyond SEM of 2 points) across the different sites (*P* = 0.90). Similarly, no difference was observed in the proportions of those who improved their gait speed (beyond the SEM of 0.06 m/s) across the four training sites (*P* = 0.47).

#### Adverse events among the training group

A total of 12 falls occurred across the four training clinics, throughout all training blocks (A-C), with more falls occurring in block B where dual task exercises were introduced. It was most common for falls to have occurred while training anticipatory postural adjustment exercises involving ball play (40% of cases) as well as during motor agility exercises. Men were overrepresented (80% of cases) among adverse events occurring during the training sessions. Six people in the training group reported non-injurious falls or stumbles during the HEP.

### Facilitators and barriers to program implementation

Seven out of ten of the CFIR sub-constructs explored in the interviews were represented in the data as influencing trainer perceptions of the program. Facilitators and barriers to program implementation could be categorized under the CFIR domains Intervention Characteristics, Outer Setting and Characteristics of Individuals. An overview of the analysis process involving coding, CFIR sub-constructs, category formation and grouping as barriers or facilitators to program delivery can be seen in Additional file 3.There was no evidence in the transcripts that CFIR sub-constructs from *Inner setting* – Evidence strength and quality; Compatibility and Implementation climate – had positive or negative influences on trainer perceptions of program delivery.

#### Perceived facilitators of program implementation

The *disease-specific nature of the program* and it sole focus on balance was considered to fill an existing gap in terms of patients’ needs – disease and symptom-specific group training. Trainers expressed how existing forms of training tended to be more general in nature, and targeted a wider spectrum of neurological diagnoses, while also combining balance with cardiovascular or muscle strength training elements. Additionally, the specific and progressive focus on balance facilitated delivering a higher level of challenge, thus enabling those at mild levels of impairment to benefit from group training.

*That it (HiBalance) is specifically targeted towards balance training, a lot of other training focuses on strength and mobility where maybe balance has been a smaller part … the specific focus on balance means that you can zone in on one area and really train it hard.*

Trainer at Clinic 1.

Trainers described how a *treatment frequency* of twice a week over 10 weeks exceeded the more standard once weekly programs already in practice at their clinics. Program frequency was considered a facilitating factor for successful implementation as it enabled therapists to attain a more in-depth knowledge of each patient’s balance capacity, while also allowing patients to gain a better understanding of their capacity. Trainers also perceived it easier to expose all patients to a sufficiently high level of balance challenge or intensity – and therefore maintain program fidelity – when participants had mild as opposed to moderate levels of balance impairment.

*Then it’s really positive to get to meet them (patients) so many times, because you really do get to know them, and because they also get the chance to get to know themselves, sometimes by doing extreme types of exercises.*

Trainer at clinic 2.

Trainers described the program as allowing for *professional autonomy within the schematic structure,* whereby exercises were not pre-set but needed to be constructed and progressed. They perceived this feature as something that encouraged creativity and ingenuity. The program structure was discussed as advantageous in two ways, firstly in providing a reassurance that all important elements would be targeted, and secondly, as the program does not consist of set exercises this allowed trainers the opportunity for professional autonomy. That exercises shifted in focus during alternate weeks was experienced as a facilitatory factor to effective delivery as it required trainers to ‘constantly re-think their approach’ to planning the training sessions.

*Because it (HiBalance) is designed and so carefully thought through, it feels as if you can’t miss any important parts of balance … it’s easy otherwise if you can improvise completely freely that you can forget a certain part because you chose those exercises you like the best, it’s a reassuring feeling.*

Trainer at clinic 1.

Having completed one entire 10-week program gave trainers greater confidence in their ability to deliver the programs core components. This *increased self-efficacy* gave them greater certainty when stressing the importance of focusing on exercise quality as opposed to intensity during the initial two weeks. Trainers also described having gained perspective on the rate of progression of exercise challenge over time.

*The difference was that we felt a little more, more confident about how we could handle everything. We knew how it would end, if you know what I mean, we know in which direction we were going. We didn’t know that the first time, but the build- up … we were much more sure about where we were heading. We felt like ─ now we are going to really drum this in, now we are going to stick to this ─.*

*because we knew that soon it would get much more difficult.*

Trainer at clinic 4.

#### Perceived barriers to program implementation

Trainers perceived *cognitive impairment* as a barrier for patients to benefit from the group sessions. They also discussed the limitations of standard physiotherapy assessment, which does not incorporate an objective assessment of cognition, when trying to establish a person’s suitability to group training. Trainers were mindful of the risk that people who performed poorly during cognitive dual-tasks could feel exposed or vulnerable in front of other group members. To offset this potential vulnerability, trainers proposed pairing patients of equal dual-task capacity.

*I remember how when we did the cognitive exercises the first time, we realized how we really had to group two (patients) at a time who were around the same level, because otherwise there could be one person that really shined at the task, and one person that didn’t, you know … you didn’t want anyone to feel like they stood out in any way.*

Trainer from clinic 2.

For similar reasons, *heterogeneous patient groups* in terms of balance capacity were considered a barrier to maintaining fidelity to program core components. If one person’s capacity differed largely from others, due to either low or high levels of balance impairment, this was considered a hinder to achieving a sufficiently high level of challenge for all members of the group.

*If someone stands out too much, because they are either too good or too bad, well then it gets very difficult to challenge them, if they have poor balance then you need to point mark them and then you lose a certain flow.*

Trainer at clinic 4.

In accordance with this, trainers perceived that homogeneity in relation to balance capacity facilitated a better group dynamic among participants. An additional barrier that emerged during interview analysis regarded patients with *impaired balance reactions*. Trainers suggested that, in the interest of patient safety when performing highly challenging exercises, people with a total absence of balance reactions should be not be included in this program.

Trainers’ difficulties in choosing a suitable *initial level of exercise challenge* was a recurring theme*,* as were difficulties determining an adequate weekly rate of exercise progression. This was especially the case when choosing cognitive dual-task exercises where it was difficult to choose an exercise that challenged patients at an individual and even more so at group level.

*I thought that it has been hard to understand which level to start at, that was really tricky. Like just how easy or difficult it was supposed to be in the beginning, because you knew that you were supposed to step up the challenge gradually.*

Trainer from clinic 3.

The *initial two weeks* of the program were perceived as somewhat repetitive and trainers felt a need to advance the exercises more than the program structure indicated. They were conscious to avoid patients feeling bored or unmotivated with the program in the early stages. This perceived difficulty was grounded in difficulties *maintaining specificity* when choosing exercises that targeted one particular balance sub-component during Block A. It was perceived as easier to construct complex exercises targeting several domains than to streamline tasks to target one specific balance component at a time. Trainers commonly perceived that maintaining fidelity to exercise specificity during Blocks A and B a greater challenge, than when all components could be targeted simultaneously in the final block of the program.

*We felt as if the first couple of weeks were too easy and that we felt to steered in the different areas, sometimes we would have liked to progress the exercises at a faster rate … and then it’s also the case that the patients want to push forward as well, they want to constantly increase the difficulty level, perhaps they thought it was too easy in the beginning.*

*Trainer from clinic 2.*

#### Trainer suggestions for program adaptation

- Adding lack of protective fall reflexes (0/6 points on the Balance Reaction sub-component of the miniBESTest) as an exclusion criterion to the program.
- Reducing the initial two-week block to one week in order to start challenging participants earlier on in the program.
- Adding basic balance exercises to the HEP and planning a third session during week one to allow for instruction and individual adaptation of the HEP.
- Setting individual goals with patients during inclusion to the groups in order to mirror clinical practice.
- Running parallel training groups for patients at mild and moderate levels of impairment, could benefit maintain fidelity to the highly challenging aspect of the program.
- Using exercise ‘stations’ when the gym space is small, so that patients of similar balance capacity can be paired. That way high levels of challenge can be achieved, and safety ensured by supervising at a particular station rather than one particular person.

## Discussion

Findings from this process evaluation show that the HiBalance intervention was delivered with very high fidelity to the programs core components. Trainers planned and delivered exercises in line with the structure intended by the research group, in all except for 4% of the sessions. Evidence for fidelity in program delivery is further supported by participant responsiveness to the programs core components, whereby a strong majority of them reported it to be challenging, progressive and specific in nature. Additionally, we find no evidence, in terms of dose delivered, dose received, or site-specific training response, to suggest that program delivery was more or less effective at any particular site, despite a variation in the nature of the clinics and trainers or varied recruitment strategies.

Qualitative findings revealed that trainers perceived lower cognitive reserve as a main threat to challenging patients at a high level during the sessions, in particular during dual-task exercises. There is extensive support for the fact that PwPD have difficulties performing dual-task exercises [21], possibly due to reduced attentional resource capacity [22]. Nevertheless, PwPD can improve their performance through training dual-task paradigms [23] [24], although reports differ regarding those most likely to benefit from integrated training techniques. Whereas participants with lower cognitive scores in the HiBalance efficacy trial were more likely to improve balance [25], others report that higher cognitive reserve better predicts positive response to dual-task training [24]. These findings however are based on cognitively unimpaired participant samples, unlike in this study where mild cognitive impairment cannot be ruled out, as cognitive testing was not performed prior to inclusion. Currently, there is insufficient evidence in the literature to enable clinicians to individually tailor dual-task training paradigms to a patient’s baseline motor-cognitive performance [26]. If trainer proficiency in dual-task techniques are increased however, they may perceive fewer barriers in this area. Trainers described, for example, initial tendencies to introduce cognitive dual-task exercises that exceeded patients’ capacity. This highlights the importance of educating trainers on how the degree of attentional load relates to the complexity of the dual-task exercise – tasks involving backwards repetition of digits and subtraction appear to place greater demands on motor performance than tasks involving word recall or forward number repetition [27]. Clinicians’ reluctance to include people with lower cognitive reserve could also be a reaction to ethical dilemmas they experienced when specific participants stood out as having a poorer cognitive capacity during group exercises. Such situations could be avoided by designing dual task exercises that are reported solely to the trainer, as opposed to other group members.

Trainers expressed uncertainties determining the appropriate initial level of balance challenge. Difficulty in quantifying the level of balance challenge is previously acknowledged among specialist clinicians [28]. Additionally, a systematic review of 148 balance training interventions found no reports of use of a validated instrument to rate the intensity of balance challenge [29]. The validation of balance intensity scales is in its infancy [30] and the added advantage of introducing such measures need to be weighed against existing clinical time constraints. Nevertheless, use of validated scales could serve a dual purpose by aiding clinicians to pinpoint appropriate starting levels of challenge, while also allowing patients to observe potential changes in perceived exercise difficulty over time. Additionally, considering the finding that a proportion of participants reported that their balance was challenged to a small (23%), or to a very small (6%) degree, the overload of balance challenge principle was not achieved in all cases. Study trainers suggested that a recruitment process aimed at forming more homogenous groups in terms of balance capacity, could enable more highly challenging group exercises to be designed and implemented.

Interestingly, all trainers in the current study reported challenges of maintaining specificity when designing exercises targeting isolated balance sub-components. That planning ‘simpler’ exercises was perceived as more challenging than designing complex exercises encompassing several balance components is somewhat surprising, considering that physiotherapists are accustomed to working autonomously and adapting programs to patients’ capacity [28]. This finding possibly reflects how physical therapists are more accustomed to focusing on complex functional exercises, which is in line with guidelines promoting ‘goal-based’ or ‘task specific’ training in neurological rehabilitation [31, 32]. Additionally, the five sessions where fidelity was poor occurred during Block A and involved a non-specific targeting of balance sub-components. This highlights the necessity to further refine educational materials concerning exercise specificity prior to a further implementation scale-up. The addition of a measure to assess skills acquisition could also ensure systematic assessment of trainer competency to design exercises isolating specific balance sub-components.

Process evaluation findings highlight the need to further refine and develop the content and delivery of the HEP. Although a majority of participants reported being positive to carrying out home training, overall adherence was low (50%). The HEP consisted of a fixed set of exercises, with suggestions for how each exercise could be progressed during the 10-week period. Although trainers were encouraged to individually adapt the HEP to the varied functional capacity of participants at program outset, trainers reported having insufficient time to individualize or progress the HEP during the intervention period. It is possible that sub-optimal individualization, progression, and active follow-up of the HEP, lead half of participants to perceive the exercises as underchallenging or irrelevant. These factors, in combination with PD-related symptoms such as initiative-taking difficulties and apathy, may explain low adherence levels to the program, and possibly also why the HEP did not result in increased levels of habitual physical activity as reported in the outcome evaluation of this trial [10]. Although limitations of unsupervised home training are reported among older adults [33] and PwPD [34, 35], a recent systematic review provides promising evidence for how home training can effectively improve balance and gait in mild-moderate PD [36]. Trainers in the current study proposed setting individualized physical activity goals at program outset, as well as an additional session focusing on the HEP alone. Goal-setting and follow-up feedback on performance could enhance the adherence and patient-centeredness of the HEP, and are behavioral change techniques with strong support in the literature [37, 38].

PwPD in the current study reported that exercises targeting sensory integration were most challenging. In the outcome evaluation no improvement in this specific domain was observed due to a strong ceiling effect [10]. Interpreted together, these findings reinforce the need for an additional outcome measure to capture potential improvements in sensory integration that can result from the HiBalance program.

Recruitment difficulties experienced in the initial trial stages − that starting clinics did not succeed in recruiting 12 PwPD to enable randomization − reflects the difficulties of participating in a clinical trial, as opposed to difficulties recruiting 6 PwPD to HiBalance training as a part of standard care. Nonetheless, our findings show that clinics with no previous experience of holding PD-specific training would need to advertise upcoming groups by communication with patient organizations or establishing a collaboration with Neurologists in their area. Concerning our operationalized definition of Reach, with respect to the wide variation in the nature of participating clinics, the broad recruitment strategies used, as well as the variation in participant characteristics reported in the outcome evaluation [10], we consider the patient population in the current study to be broadly representative of the target group for whom HiBalance is designed.

### Strengths and limitations

The major strengths of this study lie in the application of existing theoretical frameworks – MRC guidelines and CFIR – that enabled a more systematic approach to data collection and analysis and also increases the replicability and generalizability of study findings [7, 39]. Additionally, triangulation of qualitative and quantitative data provides different perspectives on aspects of program implementation. Our current findings provide further insight and corroboration of results from the outcome evaluation which can be seen as a validation of the robustness of these findings. Several limitations should however be considered. We are unable to express Reach according to the standard definition (proportion of eligible individuals who participate the intervention), as we lack data concerning the entire PD population in the region. We are therefore unable to draw any conclusions regarding the proportion or representativeness of the population we reached. Our findings concerning the variation among study participants at study sites, require further validation in a future widescale implementation study. The application of directed content analysis involves approaching the data with an explicit and informed bias. It is therefore possible that this approach increases the likelihood of producing findings supporting the predetermined theory as opposed to opposing it [19]. Qualitative interviews with people with PD would have added to the depth of our interpretation of participant responsiveness of the program core components. HiBalance trainers perceived the training frequency (20 one-hour sessions) as facilitatory for program implementation. It is possible that these positive perceptions, of a program frequency exceeding the maximum number of visits reimbursable within the Swedish healthcare system, may not reflect the concerns of rehabilitation leaders when making decisions about program implementation. This potential barrier to implementation requires investigation at leadership level.

## Conclusion

The HiBalance intervention was delivered with high fidelity, in terms of trainer adherence and participant responsiveness to the program core components. Dose delivered and received of the group training sessions was high and people with PD reached represented our target population across all sites. Trainer perceptions of barriers to program delivery, such as, difficulty maintaining specificity to exercises and setting the initial levels challenge could be overcome by further development of program materials and on-site training. Barriers such as cognitive impairment, heterogeneous groups and impaired balance reactions could be addressed by refining guidelines for group inclusion as well as increasing trainer proficiency in dual-task training approaches.

## Supplementary Information

**Additional file 1 Table S1**. Program resource materials provided to trainers for intervention delivery.

**Additional file 2 Table S2**. Description of participating clinics where training occurred.

**Additional file 3 Table S3**. Overview of the qualitative analysis process of the investigation of the barriers and facilitators to program delivery.

## Data Availability

The datasets generated and analyzed during the current study are available from the corresponding author upon reasonable request, after ethical considerations.

## Abbreviations

PD: Parkinson’s disease
PwPD: People with Parkinson’s disease
MRC: Medical Research Council
CFIR: Consolidated Framework for Implementation Research
HEP: Home exercise program
