# Supplementary material for: Implementation of highly challenging balance training for Parkinson’s disease in clinical practice: a process evaluation
Source: BMC Geriatr. 2021 Feb 1;21:96. doi: 10.1186/s12877-021-02031-1 (PMC7852138; doi:10.1186/s12877-021-02031-1)
Supplement: Supplementary file 1 — Additional file 1 Table S1. Program resource materials provided to trainers for intervention delivery. [file 12877_2021_2031_MOESM1_ESM.docx]

**Additional Table 1.** Program resource materials provided to trainers for intervention delivery

| **Program materials** | **Format and content** |
| --- | --- |
| HiBalance *Measurement* Protocol | Standardized folder outlining: |
|  | 1. Details for study inclusion criteria |
|  | 1. Flow chart for measurement |
|  | 1. Informed consent and study information |
|  | 1. Protocol for pre-test measurement |
|  | 1. Protocol for post-test measurement |
|  | 1. Patient experience questionnaire |
|  | 1. Protocol for registering accelerometers |
| Equipment for assessment of primary outcome | 1. Tilted wooden standing board |
|  | 1. T-foam cushion |
| Physical activity  monitoring kit | Padded envelope with return postage containing: |
|  | 1. Participant information and instructions |
|  | 1. Actigraph GT3X accelerometer |
|  | 1. 7-day accelerometer participant wear diary |
| HiBalance Training Protocol | Standardized folder outlining: |
|  | 1. HiBalance theory and exercise bank |
|  | 1. Protocol forms for planning of training sessions |
|  | 1. Protocol forms for reporting of training sessions |
|  | 1. Home exercise program pamphlet |
|  | 1. Protocol forms for adverse events |
| Instructional videos | Digital video recordings:  Short video clips of program core components – exercise challenging specific balance subdomains/ dual-task components |
