# Supplementary material for: Implementation of highly challenging balance training for Parkinson’s disease in clinical practice: a process evaluation
Source: BMC Geriatr. 2021 Feb 1;21:96. doi: 10.1186/s12877-021-02031-1 (PMC7852138; doi:10.1186/s12877-021-02031-1)
Supplement: Supplementary file 2 — Additional file 2 Table S2. Description of participating clinics where training occurred. [file 12877_2021_2031_MOESM2_ESM.docx]

**Additional Table 2.** Description of participating clinics where training occurred

| Clinic Description | Clinic 1 | Clinic 2 | Clinic 3 | Clinic 4 |
| --- | --- | --- | --- | --- |
|  | University hospital  Neurological department | Geriatric hospital  Primary Care | Geriatric hospital  Primary Care | Neurological rehabilitation  Clinic |
| **Geographical location** | North-central  Inner-city | Inner-city  Stockholm | West Stockholm | South  Stockholm |
| Individual outpatient rehab visits/ year | 834 | 14666 | 11617 | 3960 |
| Number of unique patients /year | 394 | 2368 | 2140 | 263 |
| Individual outpatient rehab visits for treatment of PD /year | 370 | 250 | 44 | 1071 |
| Number of unique patients with PD /year | 75 | 53 | 42 | 63 |
| Physical therapists  Total/out-patient rehab | 7/1 | 12/9 | 12/5 | 12/2 |
| Experience of PD-specific group training | Yes | Yes | No | No |
| Experience of outpatient Neurological group training | Yes | Yes | No | Yes |
|  |  |  |  |  |
| **Training semesters** | Spring 2016 | Spring 2016 | ─ | ─ |
|  | ─ | Autum 2016 | Autum 2016 | Autum 2016 |
|  | Spring 2017 | Spring 2017 | Spring 2017 | Spring 2017 |
|  | ─ | Autum 2017 | ─ | Autum 2017 |
| **PT trainers** |  |  |  |  |
| Total during study period | 4 | 3 | 4 | 2 |
| **Participants included** |  |  |  |  |
| Total included training | 13 | 22 | 8 | 18 |
| Total included controls^1^ | 34 | 7 | 9 | 0 |
|  |  |  |  |  |
| **Recruitment process** | Internal process of referral & Advertisement | Internal process of referral & Advertisement | Advertisement  only | Internal referral process |

PD: Parkinson’s disease. PT: Physical therapist. ^1^A further six control participants were included at two ‘control clinics’ not outlined in this table.
