# Supplementary material for: Implementation of highly challenging balance training for Parkinson’s disease in clinical practice: a process evaluation
Source: BMC Geriatr. 2021 Feb 1;21:96. doi: 10.1186/s12877-021-02031-1 (PMC7852138; doi:10.1186/s12877-021-02031-1)
Supplement: Supplementary file 3 — Additional file 3 Table S3. Overview of the qualitative analysis process of the investigation of the barriers and facilitators to program delivery. [file 12877_2021_2031_MOESM3_ESM.docx]

**Additional table 3.** Overview of the qualitative analysis process of the investigation of the barriers and facilitators to program delivery

|  | **Categories** | **Meaning unit** | **CFIR Sub-construct** | **Definition** |
| --- | --- | --- | --- | --- |
| Facilitator | Disease and balance specific program | *It’s nice that it (HiBalance) is targeted specifically at this diagnosis…and that it is specific balance training, there is already a lot of strength programs and mobility and so on* | Relative advantage | Trainers perception of the advantage of implementing the intervention versus an alternative solution |
| Facilitator | High frequency and intensity benefits those at mild disease stages | *I have a feeling that those patients with Parkinson’s whose symptoms haven’t progessed so, that they have a hard time finding the right forum to ventilate their thoughts and ideas as well as really train. It often feels as if a lot of training that is out there is more targeted towards those where more pronounced balance problems* | Patient needs and resources | The extent to which patient needs, as well as barriers and facilitators to meet those needs, are known and prioritized by the organization. |
| Facilitator | Autonomy within a structure | *I like that you have a skeleton structure in which you still have to make decisions in a lot of ways, dependent on the type of group you have. It’s nice to have a framework because it’s so easy to fall back on your usual favorite exercises* | Adaptability | The degree to which an intervention can be adapted, tailored, refined, or reinvented to meet local needs. |
| Facilitator | Certainty to teach the core elements increase with experience | *The second time we held the program it was easier to convince patients of the importance of repeating simple exercises in the beginning* | Self-efficacy | Trainers’ belief in their capabilities to execute courses of action to achieve implementation goals. |
| Barrier | Maintaining specificity | *During the first weeks it difficult to keep the exercises simple* | Complexity | Perceived difficulty of implementation |
| Barrier | Absence of fall-protective reactions | *You should maybe not include a patient to this group if they don’t have any fall-protective reactions…it’s hard to test because there is a lot of trust involved in that assessment, but when you notice they are lacking I think you should advise against this program* | Patients’ needs and resources | The extent to which patient needs, as well as barriers and facilitators to meet those needs, are known and prioritized by the organization. |
| Barrier | Difficult to ascertain the initial level of balance challenge | *I think it was difficult to understand which level were supposed to start at, that was tricky. Like how easy or difficult to set it, because we know that there should, where to start…. Add sth. about the pamphlets showing more advance exercises* | Access to knowledge and information | Ease of access to digestible information and knowledge about the intervention and how to incorporate it into work tasks. |
| Barrier | Heterogeneous patient groups in terms of balance | It is difficult when they (patients) are very uneven in what they can do. If one person needs to be followed and the others are clearly better, then you can’t really lift the difficulty level for the entire group | Knowledge and Beliefs about the intervention | Trainers attitudes toward and value placed on the  intervention and principles related to the intervention |
| Barrier | Low cognitive reserve restricts participation | *We have had some people who were too cognitively impaired, and that becomes almost the biggest problem. It just is, they come late and they have difficulty following instructions…* | Knowledge and Beliefs about the intervention Low cognitive reserve restricts participation | As above |
| Barrier | Low cognitive reserve restricts participation | *We have had some people who were too cognitively impaired, and that becomes almost the biggest problem. It just is, they come late and they have difficulty following instructions…* | Knowledge and Beliefs about the intervention Low cognitive reserve restricts participation | As above |
